# Same-gender differences in perioperative complications and transfusion management for lower limb arthroplasty

**DOI:** 10.1186/s12891-023-06788-x

**Published:** 2023-08-16

**Authors:** Julian Koettnitz, Filippo Migliorini, Christian D. Peterlein, Christian Götze

**Affiliations:** 1 Department of General Orthopaedics, Auguste-Viktoria-Clinic Bad Oeynhausen, University Hospital of Ruhr-University-Bochum, Am Kokturkanal, Bad Oeynhausen, 32545 Germany; 2 Department of Orthopaedics and Trauma Surgery, University Clinic Aachen, RWTH Aachen University Clinic, 52064 Aachen, Germany

**Keywords:** Complications, Blood transfusion, Gender differences, THA, TKA

## Abstract

**Introduction:**

Total hip (THA) and knee arthroplasty (TKA) are surgical interventions for patients with primary and posttraumatic osteoarthritis. The present clinical investigation compared gender differences in THA and TKA.

**Methods:**

Data from 419 patients following primary THA and TKA were collected. The occurrence of systemic and surgery-related complications, the units of blood transfused, and the change in Hb were investigated. Hb was collected preoperatively and at 1, 2, 4 and 7 days postoperatively. Statistical analysis was performed using the software IBM SPSS 28.

**Results:**

There was no significant difference in surgery-related and general complications in men between THA and TKA. A significant difference between THA and TKA in systemic complications in women was observed. No significant difference between THA and TKA in related to surgery-related complications was evidenced. In men, no difference in Hb progression was observed. In women, a significant Hb drop was evidenced (*p* = 0.03). The rate of blood transfusion units in women was significantly greater in TKA than in THA (*p* = 0.001). No statistically significant difference was observed in men in the rate of transfusion between THA and TKA.

**Conclusion:**

Perioperative care should be organized differently for women and men. Furthermore, a differentiation between the procedures for each sex could prevent the occurrence of perioperative complicated courses.

## Introduction

Osteoarthritis is a joint disorder with increasing prevalence in developed and developing countries. Some risk factors, including age, gender, obesity and diet are well-known [1]. Total hip arthroplasty (THA) and total knee arthroplasty (TKA) are milestones in the treatment of end stage osteoarthritis, restoring joint biomechanics and symptoms [2–4].

Gender difference in the outcomes following orthopaedic surgery is controversial and the demand for further studies is growing [5–7]. Previous comparative clinical investigation showed that women are more prone to complications, required a greater rate of blood transfusion units, and have worse outcomes [8–12]. To our knowledge a same-gender analyse and the comparison of differences in both gender for lower limb arthroplasty has not been performed. Therefore, this clinical investigation examined whether a gender difference in the outcome undergoing primary THA and TKA and a same-gender difference between procedures exists. The rate of blood transfusion units, surgery-related and systemic complications, and the haemoglobin (Hb) drop in THA and TKA were compared.

Through this analyse gender differences and same-gender differences for both procedures were shown.

## Methods

### Study design

The present study was performed according to the Strengthening the Reporting of Observational Studies in Epidemiology (STROBE) [13] and conducted at the Department of Orthopaedic Surgery of a University Hospital in Germany. The study was conducted in accordance with the Declaration of Helsinki and approved by the Ethics Committee of RUB-Bochum University, HDZ Bad Oeynhausen, (ID: 2020–680; 2018–625-f-S).

### Study protocol

Data from patients who underwent primary THA or TKA during 2017 and 2018 were retrieved. Data were retrieved using Pegasos 7 (Nexus Marabu GmbH, Berlin) and collected in Microsoft Excel (Microsoft Corporation, Redmond, US). The following data were collected at admission: age, sex, side, body mass index (BMI), length of hospital stay and American Society of Anaesthesiologists physical status (ASA). The ASA classification counts from 1–6 (normal health, mild, severe, severe with life-threatening conditions, moribund diseases and brain dead) [14]. The following data were collected during the hospitalization: preoperative and postoperative haemoglobin (Hb), the incidence of systemic and surgical complications, and the frequency of blood unit transfusions. Systemic complications included pulmonary, cardiac, urogenital, and neurologic complications, as well as electrolyte imbalances and exceptional laboratory parameter changes. Surgical-related complications included early infections, neurologic disorders, fractures, and ligament damage. If patient data was not accessible, the patient was excluded from the present investigation.

### Eligibility criteria

All patients undergoing primary uncemented THA or cemented TKA were retrieved and their eligibility was assessed. The inclusion criteria were: (1) Patients with primary and secondary osteoarthritis, (2) Patients aged between 61–79 years, (3) accessible patient data, (4) implantation. The exclusion criteria were: (1) revision surgery, (2) any blood abnormalities, (3) pregnancy, (4) peripheral arteriovenous or neurologic ailment (5) complete data lack of patient information.

### Perioperative management

The arthroplasties were done with Smith & Nephew Genesis II CR/PS, Journey II BCS/CR (S&N GmbH, Londo, UK) or Zimmer Biomet Fitmore stem and Alloclassic/ Allofit cup system (Zimmer Biomet, Warsaw, US). For the TKA a standard medial parapatellar approach, for the THA an anterolateral approach was used. After Surgery patients with THA got diclofenac 75 mg for 10 days to prevent heterotrophic ossification. A stationary or ambulant rehabilitation was organized prior to a hospital release.

### Blood unit supply

Indication for the blood units transfusion was according to the restrictive Cochrane guidelines: Hb-levels over 8,0 g/dl no transfusion, between 7 to 9 with concomitant clinical symptoms such as dizziness, nausea, malaise or loss of appetite and Hb-levels under 8 g/dl indicated transfusion [15].

### Statistical analyses

All statistical analyses were performed using the software IBM SPSS version 28 (IBM, Armonk, US). Metric scaled data were analysed by mean, standard deviation, and variance. Nominal, dichotomous data were analysed by Fischer’s exact Test. Age, BMI, length of hospitalization, length of intensive care stay, pre- and postoperative Hb and frequency of transfusion were listed metrically. Sex, systemic and surgical complications were listed nominally. ASA score and preconditions were listed ordinally. For the analysis of metric and nominally scaled variables, the T-test for independent samples, Variance-analyses, the Levene test, and the Welch tests were used. Cohen`s d (small 0.20; medium 0.50; large 0.80) and 95% interval were used as effect sizes. The effect size used was phi (small 0.10; medium 0.30; large 0.50). The significance level was set two-sided with α = 0.05.

## Results

### Recruitment process

**Fig. 1:**
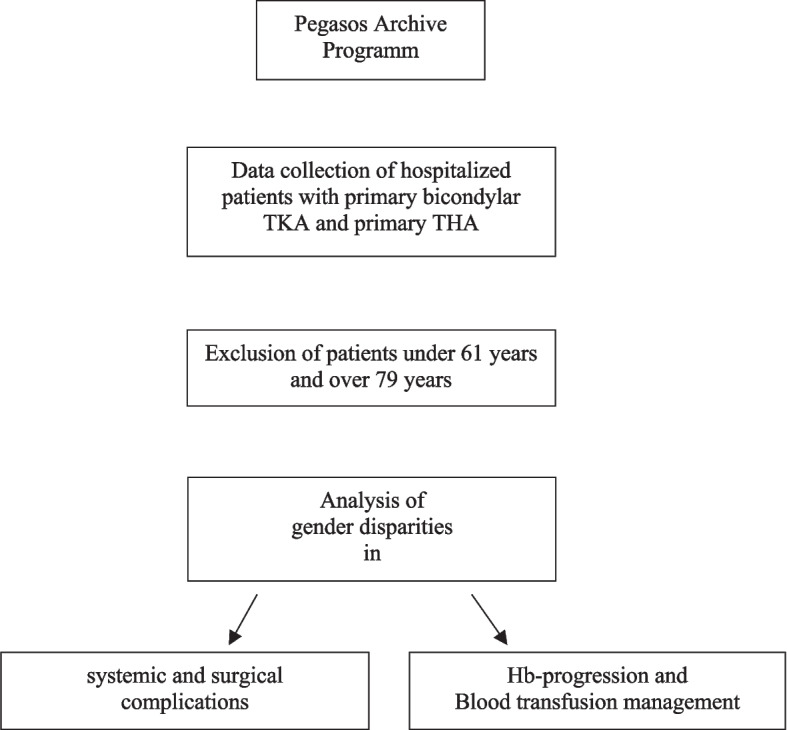
Workflow

Data from 750 patients with primary cemented knee arthroplasty and primary uncemented hip arthroplasty from 2017–2018 were retrieved. 270 patients were excluded as they were younger than 60 years or older than 80 years. 61 patients were excluded as they received partial knee arthroplasty. Finally, data from 419 patients were collected: 220 THA and 199 TKA (Fig. 1).

### Patient demographics

Data from 419 patients were collected. 39.9% (167 of 419) were men and 60.1% (252 of 419) were women. In the TKA group, there were 39.7% (79 of 199) men and 60.3% (120 of 199) women. In the THA group, there were 40% (88 of 220) men and 60% (132 of 220) women.

The mean BMI in men was 29.10 ± 4.5 kg/m^2^ and 29.30 ± 5.7 kg/m^2^ for women. The mean age of men was 70.67 ± 5.4 years and 71.31 ± 5.2 years of women. The mean ASA-Score in men was 2.1 ± 1.7 and 2.08 ± 1.7 in women. The length of the hospital stay was 10.2 ± 3.7 days in men and 10.3 ± 2.7 days in women (Table 1).

**Table 1:** Patient demographics; significance in *p*-value m = men, w = women; LOS = length of stay

Demographics (m/w)	*p*-value
Gender	0.001
BMI	0.506
Age	0.220
ASA-score	0.510
LOS	0.933

There was an overall of 3.5% (6 of 167) surgery-related complications in men and 3.1% (8 of 252) in women. There was an overall of 3.5% (6 of 167) systemic complications in men and women 13.0% (33 of 252) in women. These data are shown in greater detail in Tables 2 and 3.

**Table 2:** Systemic complications of men and women after lower limb arthroplasty

Systemic complications	Men (*n* = 6)	Women (*n* = 33)
**Pulmonary:**	**THA TKA**	**THA TKA**
-Pneumonia	- 1	1 3
-Bronchitis		- 1
**Cardiac/Vascular:**		
-Myocardial infarction		1 -
-Atrial fibrillation		- 1
-Venous Thrombosis		1 2
**Urogenital:**		
-urinary tract infection	1 -	2 11
-acute renal insufficiency		
**Other Infections:**		
-Erysipel		- 1
-Phlegmon	- 1	
-other infections	- 1	- 1
**Persistent Electrolyte imbalances**		
-Hypokalemia		- 7
-Hyponatremia	- 1	
**Neurological:**		
-Transitory psychotic syndrome	- 1	- 1

**Table 3:** Surgery-related complications of men and women

Surgery-related complications	men (*n* = 6)	women (*n* = 8)
**Surgery-associated infections**	THA TKA	THA TKA
-Wound healing disorder	3 -	2 2
- Periprosthetic infection		- 1
**Neurologic disorders**		
-Foot lifter weakness		- 1
-Paraesthesias	2 -	1 -
**Postoperative Fractures**		
-Periprosthetic fracture		
**Disorders of ligaments**		
-Internal ligament overstreching		
-Rupture of tendons	- 1	- 1

### Complications

There was no significant difference between men in THA and TKA in complications. A significant difference between THA and TKA in systemic complications in women was observed. No significant differences between THA and TKA in related to surgery-related complications were evidenced (Table 4).

**Table 4:** Systemic and surgery-related complications between men in THA and TKA group and between women in THA and TKA group

**Systemic complications between THA and TKA**	**phi**	**p**
Men	0.101	0.252
Women	0.274	0.001
**Surgery-related complications between THA and TKA**	**phi**	**p**
Men	0.05	0.721
Women	0.05	0.484

### Hb drop

**Fig. 2:**
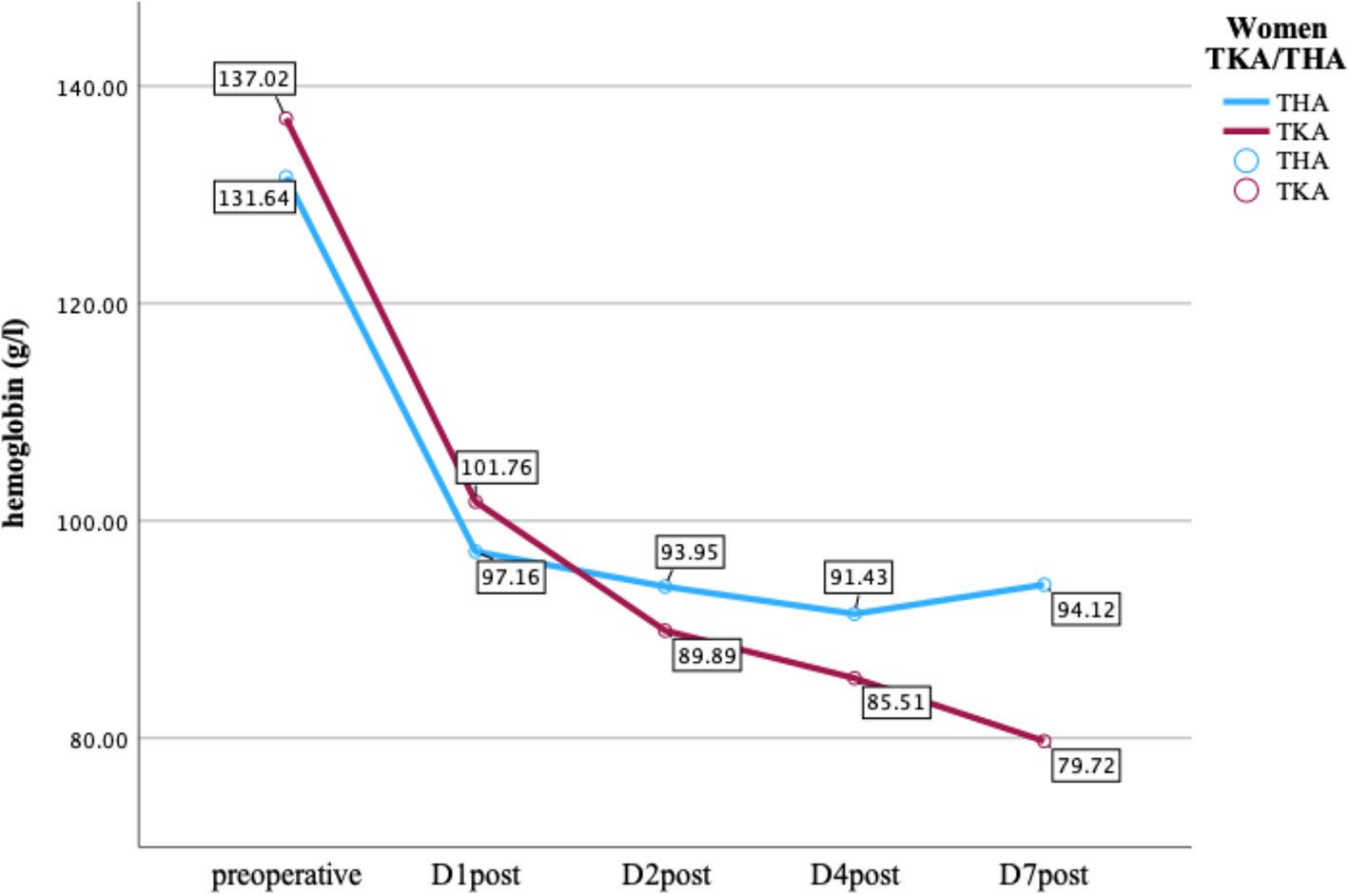
Hb progression women; THA = total hip arthroplasty; TKA = total knee arthroplasty

**Fig. 3:**
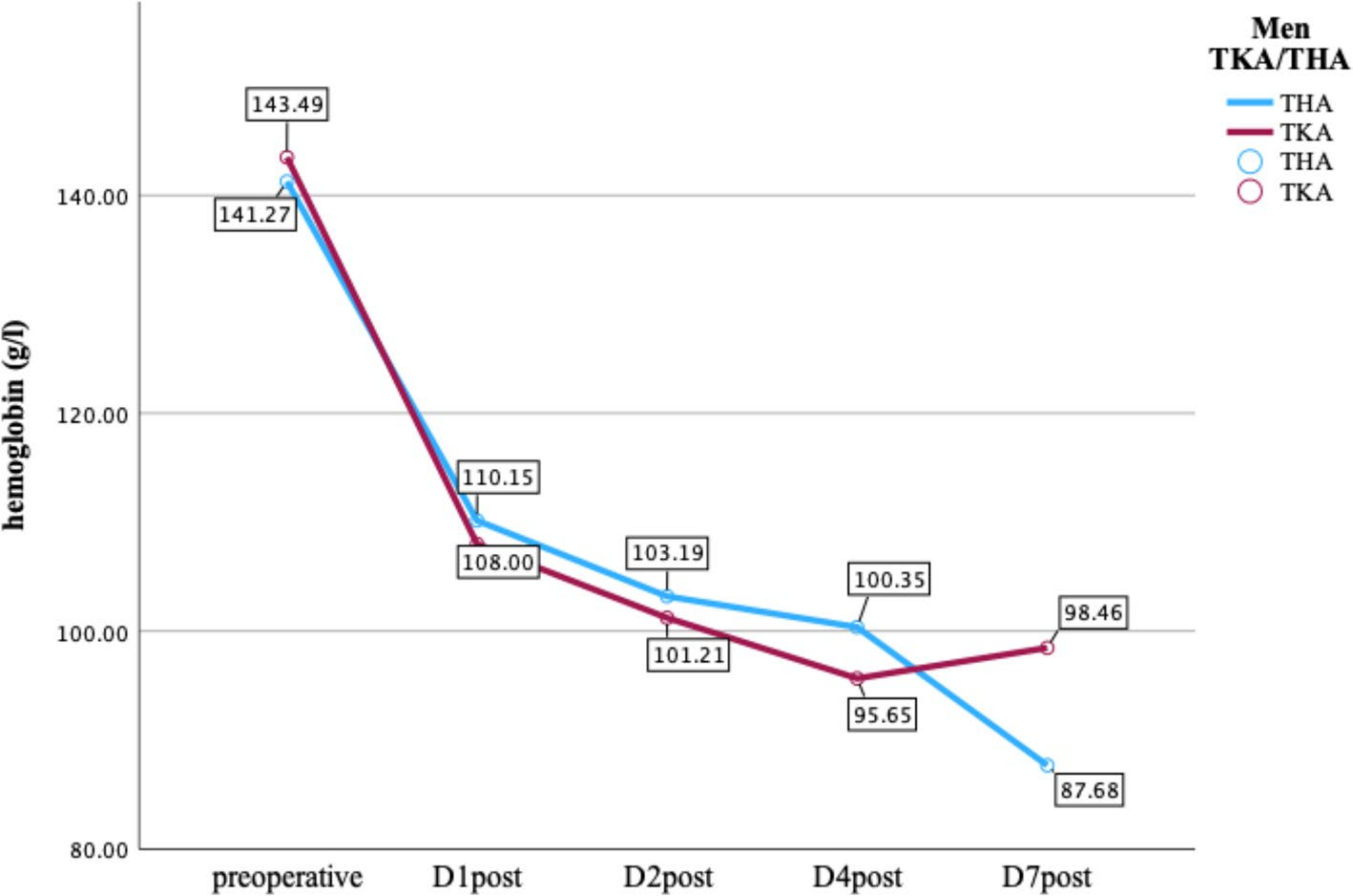
Hb progression men; THA = total hip arthroplasty; TKA = total knee arthroplasty

Hb was collected preoperatively and at POD (postoperative day) 1, 2, 4 and 7 after surgery. The mean preoperative Hb in men and in women was 142.10 ± 20.17 g/l and 135 ± 10.67 g/l, respectively. At POD 7, the mean Hb in men and for women was 91.99 ± 35.6 and 85.77 ± 37.09 g/l, respectively. In men, no difference in Hb progression was observed. In women, a significant Hb drop was evidenced (*p* = 0.03, d = 0.424). These data are shown in greater detail in Figs. 2 and 3.

### Blood units transfused

The rate of transfused blood units in women was significantly greater in TKA (1 unit *n* = 15; 2 units *n* = 10; 3 units *n* = 2) than in THA (1 time *n* = 5; 3 times *n* = 1) (*p* = 0.001, phi = 0.287). No statistically significant difference was observed in men (TKA: 1 unit *n* = 9; 2 units *n* = 2; THA: 1 unit *n* = 3; 2 units *n* = 1). Blood unit transfusions in women were more often in TKA (*n* = 35) group than in THA (*n* = 8) (*p* = 0.003, phi = 0.287). No difference was observed in the rate of transfusion in men between TKA (*n* = 10) and THA (*n* = 8) groups (Table 5).

**Table 5:** Hb requiring transfusion between men in THA and TKA group and between women in THA and TKA group

**Hb requiring transfusion between THA and TKA**	**phi**	**p**
Men	0.164	0.055
Women	0.287	0.001
**Blood transfusion between THA and TKA**	**phi**	**p**
Men	0.144	0.689
Women	0.287	0.003

## Discussion

This study investigated gender differences in systemic and surgical complications, haemoglobin progression, and the rate of blood unit transfusions during hospitalization following TKA and THA. Women revealed differences between total hip arthroplasty and total knee arthroplasty for systemic complications, Hb progression, the rate of Hb values requiring blood transfusion and the rate of blood transfusion. Men did not show any differences.

These results are consistent with previous investigations, where women demonstrated a higher risk of readmission, reoperation, and wound infection [9, 16, 17]. In addition, Sidler-Meier et al. reviewed postoperative periprosthetic proximal femoral fractures following total hip arthroplasty and women were revealed to be a risk factor [18, 19]. Women suffer from more pain during walking with fewer distances [17]. On the other hand, women were found to be a protective factor for mortality, sepsis and cardiovascular complications and greater positive changes in walking after THA and TKA [20]. Basques et al. analysing the American National Surgical Quality Improvement Program registry found that men increased the risk for death, surgical site infection, sepsis and cardiac arrest. In contrast, women revealed an increased risk for urinary tract infection [20–22].

These findings are inconsistent with this analysis. Patel et al. and Peng et al. describe women as a risk factor for especially surgery-related complications and a longer length of stay (LOS), esp. after THA (*p* = 0.001). In this investigation, no differences could be found regarding surgical-related complications or LOS. Since Patel et al. reviewed a national register including different operation methods and Peng et al. just included 33 patients with a two-third to the one-third distribution of men and women a complete comparison to this study is not possible. The gender ratio in both arthroplasty groups was 60/40%. The procedures of TKA and THA are standardized and repetitive. This leads to safety in surgery and consequently to low surgical complication rates. Contrary, the results of Basques et al. [20–22] are like those of this analysis for the occurrence of urinary tract infections. With 5.5% urinary tract infections in the TKA group and only 0.79% in the THA group, an undeniable difference was detected. This could be related to a longer hospital stay or delayed mobilization or possible prolonged use of a urinary catheter after TKA.

For Hb progression, Cherian et al. proved only a preoperative different level. Postoperative Hb levels and blood transfusion rates from 76 women and 49 men were equal after total knee arthroplasty [23]. On the other hand, Guler et al. 2022 showed a significantly different Hb drop in men to women, but the groups were small and only ages 57–79 years were included. Previous studies demonstrated that women are at a higher risk for several complications after THA. Up to 75% higher risks for blood transfusion than men figured out [24, 25]. This is coherent with this work, where women received two-fold blood transfusions than men for both groups together. Women in the TKA group even were given 3 times more blood transfusions than in the THA group, whereas men received the same amount in both groups. TKA is a more complex surgery than minimal-invasive THA and takes significantly longer on average, which can lead to increased blood loss. Moreover, about 100 more women were in the THA group, which could be an indication that THA is less bleeding.

The study has several limitations. The retrospective design misses a straight follow-up, which depends on whether re-appointments have been made. We prevent this circumstance for the prospective study design, because a fixed follow-up was established. After six months the patients will show up for a structured re-appointment. In-hospital data sometimes lack due to missing archiving. Before 2019 only the in-house archiving system was available, afterwards the digital patient file was created. A quick view of the patient parameters is possible for further studies. As a result, in this study fixed inclusion and exlusion criterias were built to reduce statistical errors. For example, patients with missing data were excluded. The gender distribution between men and women was 60% and there were two-fold women in the THA group than TKA group. Surgery was done by more than 10 different surgeons with different skill levels, which could influence the data. A further limitation was the investigation and comparision of both procedures to analyse gender differences. This could cause biases of the results, as the surgeries are different in technique, purpose and management. In futher studies the comparision of both procedures will not be done.

## Conclusion

In Conclusion, a same-gender difference in systemic complications, Hb progression and the need for blood transfusion were found. Women in the TKA group were more affected to those than women in THA. Men did not show any differences between the two groups. Additionally, women were more related to complications and blood transfusion than men. A higher focus should be placed on chronic anemia and iron deficiency in especially female patients with Hb below 13 g/dl. early therapy with e. g. may help to avoid perioperative blood transfusion. This leads to the question if the whole perioperative care should be aligned to the gender to be operated on. Further differentiation must be made between the interventions. A prospective long-interm analysis is currently operating to confirm the effect.

## Data Availability

All data are available at the corresponding author upon reasonable request.
